# A novel mRNA decay inhibitor abolishes pathophysiological cellular transition

**DOI:** 10.1038/s41420-022-01076-4

**Published:** 2022-06-07

**Authors:** Daisuke Kami, Toshimasa Ishizaki, Toshihiko Taya, Akira Katoh, Hiroyuki Kouji, Satoshi Gojo

**Affiliations:** 1 Department of Regenerative Medicine, Graduate School of Medical Science, Kyoto Prefectural University of Medicine, Kyoto, Japan; 2 Department of Pharmacology, Faculty of Medicine, Oita University, Oita, Japan; 3 Department of Cardiovascular Medicine, Graduate School of Medical Science, Kyoto Prefectural University of Medicine, Kyoto, Japan; 4 Department of Clinical Pharmacology and Therapeutics, Faculty of Medicine, Oita University, Oita, Japan; 5 Oita University Institute of Advanced Medicine, Incorporated., Oita, Japan

**Keywords:** RNA decay, Phenotypic screening

## Abstract

In cells, mRNA synthesis and decay are influenced by each other, and their balance is altered by either external or internal cues, resulting in changes in cell dynamics. We previously reported that it is important that an array of mRNAs that shape a phenotype are degraded before cellular transitions, such as cellular reprogramming and differentiation. In adipogenesis, the interaction between DDX6 and 4E-T had a definitive impact on the pathway in the processing body (PB). We screened a library of α-helix analogs with an alkaloid-like backbone to identify compounds that inhibit the binding between DDX6 and 4E-T proteins, which occurs between the α-helix of structured and internally disordered proteins. IAMC-00192 was identified as a lead compound. This compound directly inhibited the interaction between DDX6 and 4E-T. IAMC-00192 inhibited the temporal increase in PB formation that occurs during adipogenesis and epithelial-mesenchymal transition (EMT) and significantly suppressed these cellular transitions. In the EMT model, the half-life of preexisting mRNAs in PBs was extended twofold by the compound. The novel inhibitor of RNA decay not only represents a potentially useful tool to analyze in detail the pathological conditions affected by RNA decay and how it regulates the pathological state. The identification of this inhibitor may lead to the discovery of a first-in-class RNA decay inhibitor drug.

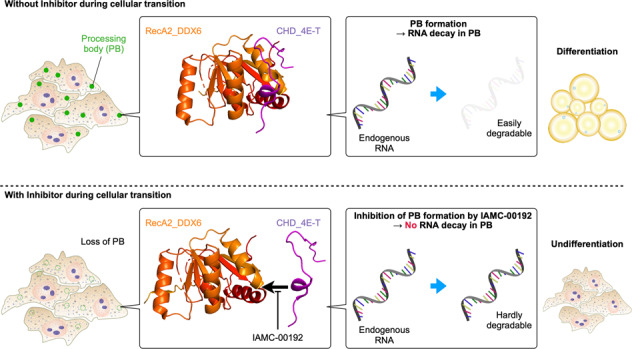

## Introduction

Cell fate upon differentiation depends on not only gene expression programs at the transcriptional level but also posttranscriptional processes, such as alternative splicing, polyadenylation, and mechanisms altering mRNA availability, including RNA decay [1]. RNA decay-dependent removal of previous cellular transcripts is a common feature of various cellular transitions, as we have previously reported that reprogramming to induce pluripotent stem cells and adipogenesis requires the eradication of the unique transcriptomes of parent cells [2, 3]. Adipogenesis is a physiological process that produces adipocytes, which are crucial for the body’s energy metabolism [4]. In contrast, ectopic adipogenesis occurs in bone [5], heart [6, 7], and skeletal muscle [8], resulting in severe functional impairment. On the other hand, the epithelial-mesenchymal transition (EMT) of cells in solid tumor metastases is an example of a dramatic change in pathogenic phenotype [9]. The ability of mesenchymal cells to acquire the capacity of active and independent migration from their tightly adherent nature to each other as epithelium allows them to form metastases at new sites located away from the primary tumor via the vasculature. Thus, phenotypic turnover is associated with a number of pathological conditions, and RNA decay is considered to be an important mechanism and target for drug discovery.

The elaborate molecular machinery involved in RNA decay has been analyzed in great detail (Fig. S1A). One of the major mRNA decay pathways initiates with the recruitment of the deadenylase complex CCR4-NOT to the 3’ untranslated regions (UTRs) of mRNAs, leading to the displacement of poly(A)-binding protein (PABP), which converts the poly(A) tail to an oligoadenylate form [10] and recruits the RNA decapping machinery [11]. Access to the 5’-terminal cap structure is attributed to 4E-T, one of the decapping machineries, which is bound to eIF4E and CNOT1, constituting CCR4-NOT, resulting in circularization of mRNA targeted for decay [12]. The physical link between CNOT1 and DDX6, a decapping activator, was also reported [13]. DDX6 functions to enhance mRNA decay through decapping [14] and constitutes processing bodies (PBs) along with 4E-T [15]. DDX6 combined with CNOT1 binds to the 4E-T enforcing bridge between the 5’-terminal and 3’-terminal in addition to the interaction of 4E-T and CNOT1 [16]. The interaction between DDX6 and 4E-T is essential for miRNA-dependent translational repression [17]. The spatial and temporal association or dissociation of these effector molecules, such as Ddx6 and 4E-T, enables fine-tuning of the transcriptome to adapt to the environmental and/or genetic cues and to maintain homeostasis in the body.

The molecular machinery of RNA quality control has been analyzed in great detail [18], and numerous proteins are involved in the formation of membraneless organelles, such as PBs and stress granules (SGs) where the fate of mRNA is regulated. Liquid–liquid phase separation (LLPS) is central to its regulation, and small-molecule compounds that specifically intervene in this mechanism have not yet been developed. Intracellular protein–protein interactions (PPIs), which are typically characterized by large flattened interfaces and hydrophobic interactions, are undruggable to small molecules and effectively inhibit binding between substances by binding concave pockets [19]. Inhibition of PPI by peptides has been proposed as an effective method to address this problem, leading to the development of some effective peptides [20, 21]. According to interactome analysis, approximately 650,000 PPIs are present in cells [22]. Although several strategies to design peptides to inhibit PPIs have been established [19, 23], most of these PPIs have not been modified or inhibited for use in basic research or drug discovery. The problems that needs to be solved for peptide drugs to intervene with PPIs include membrane impermeability and in vivo stability [24]. Although various efforts have been made, such as fusion with cell-penetrating peptides, such as TAT, the results have not yet been sufficient to push peptides into the mainstream of drug discovery [25]. Cyclic peptides that exhibit superior resistance to proteolysis and structural stability compared with linear peptides still have the issue of cell permeability. Although PPIs occur at a wide area of binding surfaces, a limited number of residues have been shown to be involved in the specificity and affinity of their binding and are referred to as hot spots [26]. A common motif found on the binding interface of many PPIs is the α-helix structure with such hot spots [27]. Compounds that mimic this set of planar side chains abrogate α-helix-mediated PPIs [28]. Such fragment-based approaches have been successfully employed to modulate PPIs and generated venetoclax, which was approved by the FDA in 2016 as a first-in-class agent that targets the anti-apoptotic protein Bcl-2 and is clinically used for the treatment of chronic lymphocytic leukemia [29]. Synthetic nonpeptidic small molecules that mimic the structure of a natural α-helix using a template projecting residue with intended arrangements have been intensively investigated [30].

In this study, we investigated whether RNA decay inhibition could inhibit adipogenesis as a physiological process and EMT as a pathological process. The binding of DDX6 and 4E-T, a core component of RNA decay, promotes the decay process by looping single-stranded RNA structures, and we decided to search for small-molecule compounds that inhibit binding at this step. DDX6 and 4E-T are structured and unstructured (intrinsically disordered) proteins, respectively, and a previous report on the interface structure indicated α-helix involvement [16]. We hypothesized that the use of peptidomimetics with cubic scaffolds (PiPiQ) [31] for such a PPI could disrupt the interaction between DDX6 and 4E-T. The development of small-molecule compounds that intervene in the RNA decay process has not been performed to date, and intervention through RNA decay in cellular transitions, including ectopic adipogenesis and cancer metastasis, is considered to have great potential as a new therapeutic target.

## Results

### Screening for an inhibitor of DDX6 and 4E-T binding

**Fig. 1:**
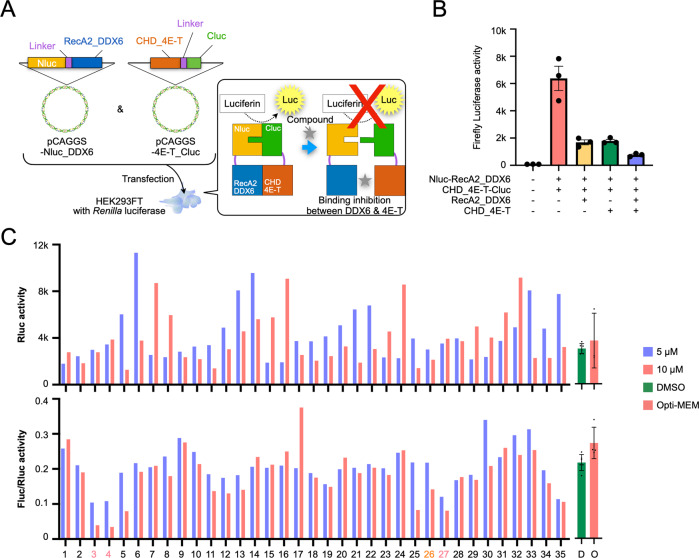
A high-throughput screening system for the discovery of small-molecule inhibitors related to RNA degradation. **A** Design of an RNA degradation inhibitor screening assay. **B** Binding and inhibition experiments of Nluc and Cluc using luciferase activity assay. **C** Cytotoxicity (Rluc) and binding inhibition (Fluc/Rluc) assessed when each inhibitor was added. D DMSO, O Opti-MEM.

It has been reported that DDX6 and 4E-T bind directly through RecA2 and the CHD region, respectively, in PBs and are involved in RNA decay [16]. Additionally, knockout of these proteins prevents PB formation. We hypothesized that inhibition of the binding of these proteins would also inhibit RNA decay, so we screened for small molecule compounds that inhibit the interaction of these proteins. To screen for such small molecule compounds, two recombinant DNA fragments were designed so that the firefly luciferase enzyme (Fluc) was split into two parts, the N side (Nluc, amino acids 1-416) and the C side (Cluc, amino acids 394-550), each of which would be a protein linked to the RecA2 region of DDX6 and the CHD region of the 4E-T binding fragments, respectively (Fig. S1B). When the two recombinant proteins are introduced into cells and RNA decay is activated, DDX6 and 4E-T bind. Thus, the N- or C-fragment of the enzyme linked to each of the target proteins yields a luciferase protein with enzymatic activity (Fig. 1A).

These fusion genes were loaded onto a forced expression plasmid DNA and transduced into Renilla (*Renilla reniformis*) luciferase (Rluc)-overexpressing HEK293FT cells, and luciferase activity was observed in the cells using Rluc as an internal control. Either RecA2 of DDX6 or CHD of 4E-T suppressed Fluc activity in genetically modified HEK293FT cells (Fig. 1B). Therefore, Fluc activity is expected to be mediated by the binding of both proteins. These plasmids and cells were used to screen 35 small molecule compounds, which were predesigned based on commonly used motifs in α-helixes. Rluc and the ratio of Fluc to Rluc were used to evaluate cytotoxicity and inhibitory efficacy, respectively. We identified four compounds (#3, 4, 26, 27) that caused a reduction in the Fluc/Rluc ratio in a concentration-dependent manner (Fig. 1C). These compounds were structurally very similar. We focused on the four compounds in the subsequent experiments.

### Inhibition of P-body formation by a small molecular inhibitor

**Fig. 2:**
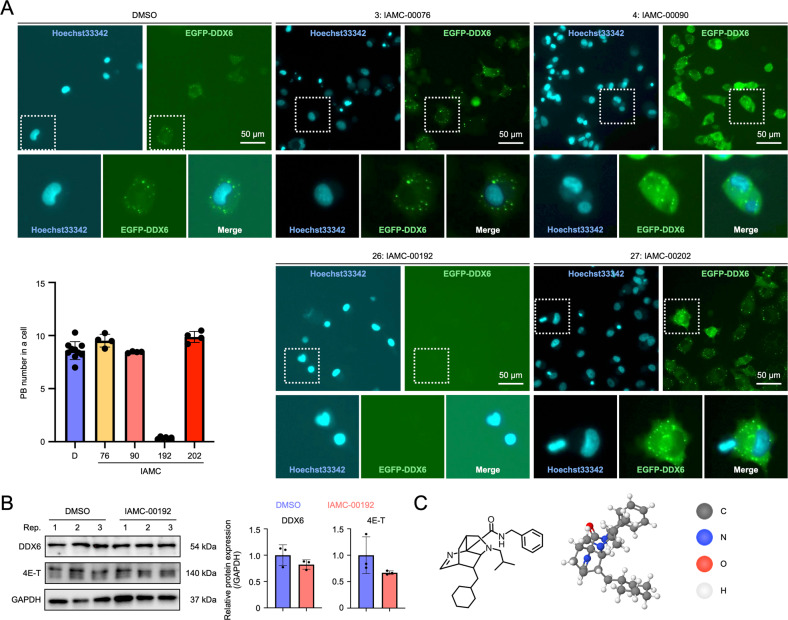
Inhibition of PB formation by the small molecule inhibitors. **A** Exposure experiments of candidate factors inhibiting RNA degradation to A549 EGFP-DDX6. GFP foci are PB. Nuclei were stained with Hoechst33342. The graph shows the number of intracellular PBs when each small molecule inhibitor was added. **B** Protein expression analysis of A549 when a small molecule inhibitor IAMC-00192 was added using Western blotting. Each graph shows the result in triplicate. **C** Structure of IAMC-00192. The molecular model on the right is the 3D structure described by MolView (https://molview.org).

We investigated whether these four compounds, which are less cytotoxic and have been observed to inhibit luciferase activity by inhibiting the binding of DDX6 to 4E-T, inhibit PB formation. A human lung adenocarcinoma cell line, A549, which possesses a significant number of PBs under conventional culture conditions, was forced to express EGFP-DDX6, which is a fusion of the PB marker DDX6 and EGFP, using a retroviral vector. PBs were visualized to observe the effects of the candidate compound in PB formation. The results showed that PBs did not form in the cytoplasm of cells only when treated with #26 (IAMC-00192) [32] (Fig. 2A). DDX6 and 4E-T protein levels in the cells did not change significantly, suggesting that IAMC-00192 did not affect protein expression but rather inhibited PB formation (Fig. 2B, Fig. S2). This compound with a molecular weight of 421.63 and a chemical formula of C_27_H_39_N_3_O is depicted using 3D modeling in Fig. 2C.

### Protein–protein interaction analysis

**Fig. 3:**
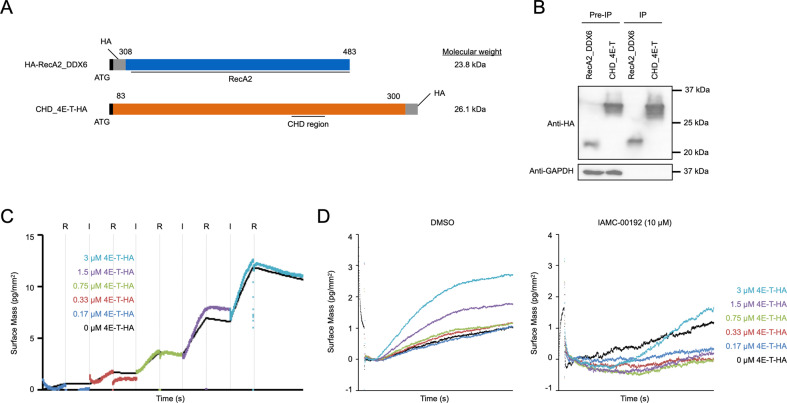
Protein-protein interaction analysis using Creoptix WAVEsystem and inhibitor candidate molecules. **A** Design of proteins for use in measuring protein-protein interactions. **B** Expression and purification confirmation of the designed protein. The protein was immunoprecipitated with HA antibody and verified by Western blotting. **C** Measurement of changes in binding strength due to changes in the concentration of CHD_4E-T protein. R Rinsing the sensor with buffer, I Injection of analyte proteins. **D** Measurement of the change in binding strength when the RNA degradation inhibitor IAMC-00192 is added.

To evaluate the binding of DDX6 to the 4E-T protein and the inhibitory ability of IAMC-00192, we measured PPI by grating-coupled interferometry (GCI) using the WAVE system (Creoptix AG). The proteins required for this WAVE system assay were designed as HA-tagged DDX6 and 4E-T (Fig. 3A), force-expressed in HEK293FT cells and clearly purified by immunoprecipitation using an HA antibody (Fig. 3B, Fig. S3). To calculate the dissociation constant (KD) between two molecules and the K_on_ and K_off_ constant rate more specifically, we performed regeneration-free kinetics using the WAVE system (Fig. 3C). First, DDX6 protein was immobilized on the surface of the sensor, and the association and dissociation of 4E-T protein at each concentration were measured continuously from low concentrations. The results showed that the association and dissociation of DDX6 and 4E-T showed values of KD = 774.3 nM, K_on_ = 3.59 × 10^2^ ± 1.33 × 10^2 ^M^−1^ s^−1^, and K_off_ = 2.78 × 10^−4 ^s^−1^, indicating that these proteins have suitable affinity, relatively gradual association and slow dissociation rate. The same assays were performed in the presence or absence of IAMC-00192 (10 µM) to evaluate its function as an inhibitor (Fig. 3D). To correct for the bulk effect of DMSO, which is the solvent of IAMC-00192, we used a buffer containing the same concentration of DMSO (0.1%) as the solvent of the inhibitor. Of note, the Y-axis was set to all zeros at the start of binding at each concentration during the data process rather than using the prescribed analysis method given the gap in the Y-axis between the association phase and dissociation phase. As a result, clear inhibition of DDX6 and 4E-T protein binding was observed in the presence of the inhibitor (Fig. 3D right) compared to the results obtained with DMSO alone (Fig. 3D left).

### Inhibition of Adipogenic Differentiation by Small Molecule Inhibitors

**Fig. 4:**
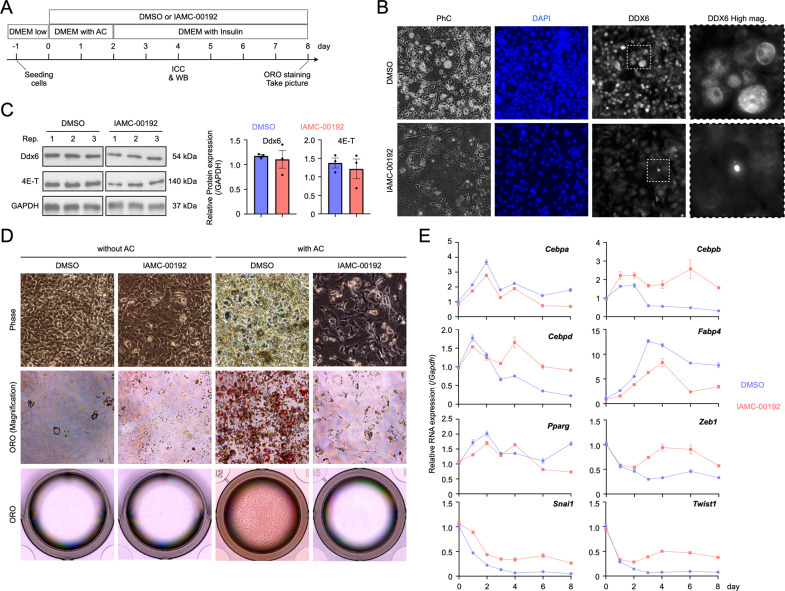
Inhibition of processing body formation during induction of adipogenic differentiation. **A** Experimental design for induction of adipogenic differentiation: DMEM low DMEM low glucose with 10% FBS, AC adipogenic-cocktail (AC) containing insulin, IBMX, and dexamethasone. ICC Immunocytochemistry. WB Western Blotting. ORO Oil Red O. **B** Immunocytochemical staining using DDX6 antibody. 3T3-L1 cells were fixed and stained on day 4 of adipogenic differentiation. **C** WB analysis of 3T3-L1 cells on day 4 of stimulation for adipogenic differentiation. The graph shows the expression ratio of each protein corrected by Gapdh protein expression level. Rep replication. **D** Phase-contrast images (top row) and ORO staining results (middle and bottom rows) of 3T3-L1 cells on day 8 of stimulation for adipogenic differentiation. The middle row is a magnified image, and the lower row is the entire image of well (12-well plate). **E** Gene expression analysis of 3T3-L1 cells stimulated by the adipogenic differentiation over time by qPCR. Design of the analysis of RNA expression in the cytoplasm and PB.

We previously reported that RNA decay in PBs is indispensable for the induction of adipogenic differentiation [2]. When DDX6 and 4E-T were inhibited by RNAi, no PBs were formed. In addition, adipogenic differentiation was arrested, and the remaining transcripts shaped the phenotype of preadipocytes. We examined whether the small molecule inhibitor we identified in this study inhibits PB formation and adipogenesis in the 3T3-L1 preadipocyte differentiation system upon treatment with an adipocyte cocktail (AC: insulin, IBMX, and dexamethasone) (Fig. 4A). Before induction, 3T3-L1 cells do not form PBs, which begin to form around Day 4 of adipogenic stimulation [2]. We compared IAMC-00192-treated and -nontreated 3T3-L1 cells and found that IAMC-00192 treatment inhibited PB formation (Fig. 4B). On the other hand, no differences in DDX6 and 4E-T protein levels were noted in these cells (Fig. 4C, Fig. S2). These results suggest that IAMC-00192 inhibits PB formation in a physiological setting without affecting DDX6 and 4E-T protein expression, and similar results were obtained in A549 cells. After 8 days of induction of adipogenic differentiation, almost no lipid droplets were observed in the experimental group treated with IAMC-00192 (Fig. 4D). On the other hand, the control group showed differentiation into adipocytes with many lipid droplets that showed strong positivity as assessed by Oil Red O (ORO) staining (Fig. S4). Gene expression of adipogenic differentiation-related factors and EMT was analyzed (Fig. 4E). *Cebpa* and *Fabp4* levels were lower in the cells treated with IAMC-00192 at all time points. *Cebpb* and *Cebpd*, which are transiently expressed in the early stage of adipogenic differentiation, were maintained by IAMC-00192. Reduced *Pparg* expression was noted in cells treated with IAMC-00192 alone for 8 days. Next, we evaluated factors associated with EMT that change with adipogenic differentiation and found that *Zeb1*, *Snai1*, and *Twist1* genes were all highly elevated from Day 3 to Day 8 in cells treated with IAMC-00192, indicating that several key transcripts for adipogenesis were maintained even after the external cue. These results suggest that the inhibitor of DDX6 and the 4E-T interaction can modulate RNA decay and intervene in adipogenesis.

### RNA analysis in P-bodies

**Fig. 5:**
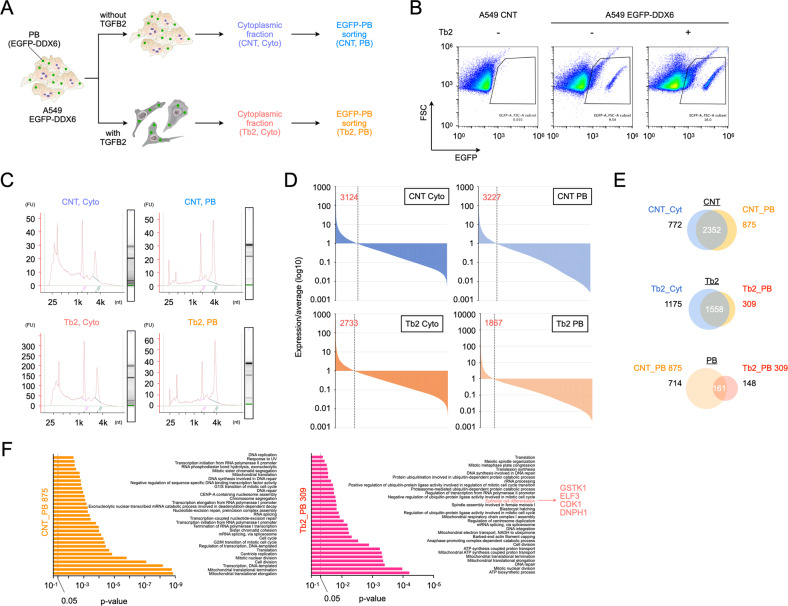
Analysis of RNA molecules contained in PB using a fluorescence-activated particle sorting. **A** The cells were A549 EGFP-DDX6, in which EGFP-DDX6 was expressed by pMXs retrovirus vector. Each cell was separated into cells with TGFB2 (Tb2) and without TGFB2 (CNT), and the cytoplasmic fraction (Cyto.) of each cell was collected, and RNA was collected. Additionally, EGFP-fluorescent PBs (PB) were isolated from the cytoplasmic fraction by FACS. **B** Representative figure for PB sorting. **C** Quality check of RNA collected by PB sorting. The evaluation was done with an Agilent Bioanalyzer (*n* = 1). **D** The exploration of gene groups whose gene expression levels in each condition are higher than the average statement value (*n* = 1). The expression levels were quantified by fragments per kilobase of exon per million reads mapped (FPKM). **E** Venn diagram for each condition. **F** Gene Ontology analysis results using DAIVD for each condition.

Endogenous RNA decay is expected to be important not only in the induction of adipogenic differentiation but also in cellular transitions, such as EMT. Therefore, we evaluated the effect of IAMC-00192 on EMT using A549 cells, a model for cellular transitions. We evaluated the RNA molecules contained in the PBs of A549 cells during the EMT process. To evaluate how inhibition of PB formation affects cellular transitions in pathophysiological conditions, we analyzed RNA molecules contained in PBs using the RNA-seq method [33]. First, EGFP-DDX6 was force-expressed in A549 cells to establish cells in which PB fluoresces with EGFP. Using these cells, RNA was collected before and after EMT with TGFB2 (Tb2) (CNT and Tb2) from the cytoplasm and PBs (Cyto and PB) (Fig. 5A). PBs were collected by FACS (Fig. 5B). The collected RNAs were analyzed by next-generation sequencing. The quality of the collected RNA was assessed, and it was found that the PB fraction contained fewer short RNA molecules than the cytoplasm (Fig. 5C). Given the lack of a common internal standard factor in this analysis, it was difficult to compare the expression levels among samples. Therefore, we decided to identify the genes that showed higher expression levels than the average expression level of each sample and analyze the common gene groups. In total, 3124 genes in CNT Cyto, 3227 genes in CNT PB, 2733 genes in Tb2 Cyto, and 1867 genes in Tb2 PB showed higher than average values (Fig. 5D). Next, there were 875 genes specific for PBs in CNTs and 309 genes specific for Tb2 (Fig. 5E). Additionally, 714 and 148 genes were specific for CNT and Tb2, respectively, in PBs. When these genes were analyzed using functional enrichment analysis, many genes related to mitochondria and cell division were found in both conditions. In addition, factors related to epithelial cell differentiation, such as *GSTK1*, *ELF3*, *CDK1*, and *DNPH1*, were found in the PB fraction with Tb2 (Fig. 5F, Dataset 1).

### Epithelial-mesenchymal transition inhibition by small-molecule inhibitors

**Fig. 6:**
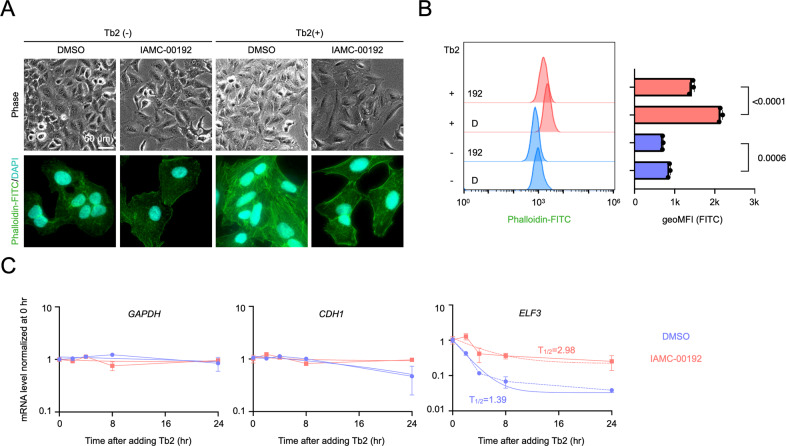
Inhibition of epithelial-mesenchymal transition by a small molecule inhibitor. **A** Phase-contrast microscopy image and fluorescence image stained with phalloidin-FITC of EMT differentiated A549 cells with Tb2 for 2 days. **B** Tb2 was added to A549 cells for 2 days, and cells stained with phalloidin-FITC were analyzed by FACS. Results of quantitative analysis of EMT-differentiated A549 stained with phalloidin-FITC by FACS. The left figure shows the FACS results, and the right graph shows the FITC fluorescence intensity of these results quantified by the geometric mean. geoMFI geometric mean fluorescence intensity. **C** Gene expression analysis in relation to EMT using the qPCR method. **D** Inhibition analysis of RNA degradation in the early stage of the induction of IAMC-00192.

IAMC-00192 inhibited the induction of adipogenic differentiation associated with EMT suppression through the suppression of RNA decay. Therefore, we evaluated IAMC-00192 using A549 and Tb2 cells, which are model cells for EMT. EMT-induced A549 cells form intracellular F-actin, which reacts with phalloidin. IAMC-00192 inhibited the formation of intracellular F-actin and drastically reduced the amount of reaction (Fig. 6A, B, Fig. S5), indicating significant suppression of EMT. These results showed that the phenotype emerged on Day 2. We thought that RNA degradation should be evaluated at an earlier stage, so we evaluated RNA degradation in the early stage (0-24 hr) of Tb2 stimulation with actinomycin D (ActD). The results showed that IAMC-00192 enhanced the stability of the *ELF3* gene and increased the RNA half-life 2.1-fold during this period (Fig. 6C). On the other hand, no significant changes in the *GAPDH* and *CDH1* genes were noted. In the EMT model, the compounds we found also suppressed RNA decay, resulting in a preexisting transcriptome, which subsequently inhibited the expression of the transcriptome characteristic of the new mesenchymal phenotype. This finding is similar to the inhibition of transcriptome expression in parental cells leading to the suppression of differentiation to mature adipocytes in adipogenesis. In both physiological and pathological processes, cellular transitions were suppressed by our compound to inhibit the RNA decay core machinery.

## Discussion

In the present study, we selected small molecule compounds that inhibit the association of DDX6 and 4E-T using the strategy of peptidomimetics and found that the compounds suppressed the elimination of transcripts encoding proteins constituting the preexisting phenotype in adipogenic differentiation and EMT of cancer cells. Additionally, PBs, membraneless organelles in the cytoplasm that are thought to be the site of RNA decay, were also reduced. Thus, differentiation induction could not occur. The lead compound in this study inhibited the binding of DDX6 and 4E-T in a concentration-dependent manner in the PPI in vitro, representing a first-in-class compound that modifies or inhibits PPI in RNA decay machinery. This study demonstrates that α-helix mimetics using PiPiQ can effectively inhibit intracellular PPI for RNA decay.

The design of peptidomimetics can be broadly classified into four categories [23]. The first categories involves the amino acid sequence of the target peptide, and the second is the peptide that is modified in various ways, including the backbone and amino acid residues. Third, the backbone is completely replaced by a small molecule, and several substitutes are projected from the scaffold to mimic the arrangement of amino acid residues in the hot spot. Finally, the compound mimics the mode of action of binding to a ligand that is not directly related to the hot spot. Methodologies to generate mimetics for various secondary structures, such as β-sheets and α-helices, have been intensively investigated. In this study, we adopt the third category for peptidomimetics, replacing the backbone with curbed alkaloid-like small molecule compounds, mimicking the spatial orientation of the R, R + 3, and R + 4 amino acid residues of the target peptide and projecting from its scaffold to a single surface of a PPI, named PiPiQ [31]. With respect to the properties of proteins, PPIs can be classified into domain-domain and peptide-domain interfaces. The latter has a unique secondary structure in the corresponding peptide, typically an α-helix structure, which is intrinsically disordered [34] and makes extensive use of several motifs, such as LxxLL [35] and FxxFF [36]. The three amino acid residues in PiPiQ were selected based on these motifs, and a library of 200 compounds was constructed for the screening performed this study. The three-dimensional structures of the DDX6 and 4E-T proteins themselves and their binding surfaces have been analyzed and are predicted to form the helix of the disordered protein 4E-T. In the attempt to create an inhibitor for PPI, designing a peptidomimetic that mimics the hot spots from the reported 3D structure is an obviously time-consuming task that requires optimization of the design while confirming the inhibitory effect on PPI. On the other hand, given the availability of a prefixed library of 200 compounds as α-helix mimetics, we thought it would be a faster to find the lead compound screening this library and ultimately discovered IAMC-00192.

Many methods for the in vitro evaluation of PPIs have been reported, including fluorescence-based, nuclear magnetic resonance-, and surface plasmon resonance (SPR)-based methods [34]. Direct labeling of proteins and peptides has been simple and widely used, but fluorescence polarization (FP) [37], which is based on the change in anisotropy value using polarized light, and fluorescence resonance energy transfer (FRET) [38], which is based on the transfer of excitation energy between two chromophores by electron resonance, have also been reported as standards for PPI detection. In this study, we constructed a competitor assay by fusing a split luciferase with each protein fragment [39] and performed the first screening to search for the inhibitor. This method is unique in that it does not require direct fluorescent labeling, antibodies, or beads and can be performed exclusively using conventional molecular biological recombination techniques. Many methods have been reported to quantitatively measure the kinetics and affinity of PPI and the efficacy of inhibitors, such as SPR [40] and isothermal calorimetry [41]. The application of SPR to PPI is based on the fact that a protein/peptide/fragment, which forms the interest of PPI, is solidified on a gold chip and excited by polarized light, causes collective oscillations and measures SPR reflectance. Technically, using a microfluidic system, changes in the SPR signal can be detected by injecting and washing out the corresponding proteins relative to the solidified proteins, and the binding constants can be derived from the association and dissociation rates [42]. The system can be used as a competitive assay by introducing a putative inhibitor into this channel [43]. In this experiment, the grating coupled acoustic wave was used to calculate the coupling constants of DDX6 and 4E-T [44], and the effectiveness of inhibition by IAMC-00192 was also confirmed. The advantage of this system is that the amount of protein required is the same as that used for expression experiments at the conventional laboratory level. Additionally, Biacore, which is based on the SPR principle, was not sensitive enough to measure this PPI in this study, suggesting that the appropriate measurement method may differ depending on the nature of the target protein.

The effective in vitro IAMC-00192 concentration discovered in this study is 10 μM, and it is still a lead compound that needs to be optimized. Using this compound as a foothold, we will need to establish its efficacy at subμM concentrations and investigate its off-target and precise allosteric effects. In addition, it is necessary to reproduce the in vitro effects in vivo as a step toward clinical use. In the postgenomic era, post-transcriptional modification has been shown to be involved in a wide variety of biological reactions, but the development of compounds to modify these processes has not progressed as expected. The discovery of compounds that inhibit RNA decay, which is one of the intracellular PPIs and is regulated by and involved in a large number of proteins through LLPS, opens up the possibility of developing various drugs that target RNA decay and provides a strategy for targeting EMT and ectopic adipogenesis. This is particularly important given the lack of treatments is currently available to treat these conditions.

## Materials and methods

### Drug screening using luciferase activity

HEK293FT Rluc cells (5 ×10^3^ per well) seeded with Nluc_DDX6 and Cluc_4E-T plasmid (20 ng per well) and Lipofectamine 3000 (Thermo Fisher Scientific Incorporated) in White/96-well Clear Flat Bottom TC-treated Culture Microplate (Corning Incorporated. Corning, New York, US). Briefly, the plasmid and lipofectamine 3000 reacted in Opti-MEM for 10 min at room temperature, mixed with trypsin-treated cells, and seeded onto 96-well plates. Eighteen hours after seeding, the cells were incubated for 48 h with exposure to the small molecule compound. Eighteen hours after seeding, the cells were further incubated with small molecular inhibitors, and 48 h later, these cells were assayed for luciferase levels using the Dual-luciferase reporter assay system (Promega Corporation) and Tecan Infinite F200 plate reader (Tecan Group Ltd. Männedorf, Switzerland) was used for measurement.

### Protein-protein interaction analysis by WAVEsystem

HA-tags fused DDX6 and 4E-T proteins were designed around the domain of RecA2 and CHD regions, respectively (Fig. S1B). This PPI was measured by WAVEsystem (Creoptix AG, Wädenswil, Switzerland). The DDX6 protein was used as a ligand and immobilized on the sensor chip by the amine coupling method using an Amine Coupling Kit (Cytiva, Marlborough, MA, United States). The 4E-T protein was used as an analyte and the PPI was measured. PBS/0.01% Tween20 was used as the running buffer. The measurements were performed in Regeneration free Kinetics mode and the various constants were calculated. A blank without DDX6 immobilization was used as a reference for non-specific binding.

### mRNA half-life measurements

For mRNA half-life measurements, transcription was blocked by actinomycin D (2.5 µg/ml) and the cells were harvested at the indicated time points using TRIzol reagent and Direct-zol RNA Miniprep Kits. Determine the mRNA decay rate by nonlinear regression curve fitting (one phase decay) using GraphPad Prism (Prism 8.4.3 software, GraphPad Prism Software Incorporated, San Diego, CA, USA) [45].

### Statistical analysis

The results are expressed as the means ± Standard Deviation (SD). The statistical significance of differences among groups was evaluated using parametric unpaired *t*-tests for bar graphs using GraphPad Prism. *P* < 0.05 was considered to indicate significance.

## Supplementary information

Supplementary information

Supplementary Figure 1

Supplementary Figure 2

Supplementary Figure 3

Supplementary Figure 4

Supplementary Figure 5

Supplementary Table 1

Supplementary Table 2

Data Set

## Data Availability

The present study’s data are available from the corresponding author upon reasonable request.
