# Supplementary material for: A novel mRNA decay inhibitor abolishes pathophysiological cellular transition
Source: Cell Death Discov. 2022 Jun 7;8:278. doi: 10.1038/s41420-022-01076-4 (PMC9174231; doi:10.1038/s41420-022-01076-4)
Supplement: Supplementary file 1 — Supplementary information [file 41420_2022_1076_MOESM1_ESM.docx]

**Supplementary Information**

**Materials and Methods**

***Cell culture***

A549 cells were purchased from RIKEN Bioresource Research Center (RCB3677, https://cell.brc.riken.jp/en/). HEK293FT cells were purchased from Thermo (R70007). These cells were cultured in Dulbecco's Modified Eagle Medium (DMEM) high glucose (FUJIFILM Wako Pure Chemical Corporation, Tokyo, Japan) with 10% fetal bovine serum (FBS) under 5% CO_2_ conditions. Induction of EMT differentiation in A549 was analyzed 2 days after the addition of Recombinant Human TGF-beta 2 Protein (302-B2-002/CF, R&D Systems, Incorporation, Minneapolis, MN, USA) at 5 ng/ml under the presence of DMEM with 5% FBS.

Preadipocyte 3T3-L1 was purchased from American Type Culture Collection. The culture conditions were DMEM low glucose (FUJIFILM Wako Pure Chemical Corporation) with 10% FBS under 5% CO_2_ condition at 37ºC. For induction of adipogenic differentiation, the cells were cultured in DMEM high glucose with 10% FBS under 5% CO_2_ condition at 37ºC and adipogenic differentiation inducers, dexamethasone (1 µM), insulin (5 µg/ml), 3-isobutyl-1-methylxanthine (IBMX, 0.5 mM) (Merck KGaA, Darmstadt, Germany), were used^1,2^. Briefly, cells were cultured in DMEM with adipogenic differentiation inducers for 2 days and then in a medium containing only insulin. The media was exchanged every 48-72 hr throughout all experiments.

***Plasmid construction and establishment of gene-modified cells***

All plasmid DNA was done under the permission of the Genetic Recombination Committee of the Kyoto Prefectural University of Medicine. RecA2 region on DDX6 (308 aa - 483 aa) and CHD region on 4E-T (196 aa - 257 aa) were cloned from RNA of A549 cultured cells, and Firefly N-terminal luciferase (Nluc) and C-terminal luciferase (Cluc)^3^ conjugated sequences were synthesized and cloned by GENEWIZ. Each sequence was inserted into the CAGGS promoter plasmid (pCAGGS). *Renilla* luciferase (Rluc) was cloned from pRL-CMV (Promega Corporation. Madison, Wisconsin, U. S.) and inserted into a pLV lentivirus vector. Cells infected with Rluc were single-cell cloned using a cell sorter SH800S (Sony Corporation, Tokyo, Japan). The proliferating cells were analyzed for Rluc gene expression using qPCR, and the cells with high expression levels were defined as HEK293FT Rluc cell line. The sequence of EGFP fused to the N-terminus of human DDX6 was inserted into the retroviral vector pMXs containing a puromycin resistance gene (RTV-012, Cell Biolabs Incorporated., San Diego, CA, USA). The pMXs retroviral vector carrying DDX6 with EGFP was infected into A549 cells and infected cells (A549 EGFP-DDX6) were selected in the presence of puromycin (Thermo Fisher Scientific Incorporated) and high expression EGFP sorting using SH800S. Each plasmid sequence is available in Supplemental Table 2.

***Protein purification using HA tag***

Each hemagglutinin (HA)-tagged protein was introduced into HEK293FT using Lipofectamine 3000, and after 2 days, all HA-tagged proteins were collected using magnetic beads coated with anti-HA antibody. Briefly, cells (5 × 10^5^) were seeded in a 100 mm dish (Corning Incorporated). The next day, after replacement with 10 ml of fresh medium, 3 µg of plasmid and Lipofectamine 3000 were reacted in Opti-MEM for 10 min at room temperature and mixed into the culture medium. After 2 days, the cells were collected using a cell scraper and eluted with RIPA (FUJIFILM Wako Pure Chemical Corporation). The eluted proteins were purified using the HA-tagged Protein Magnetic Purification Kit (MEDICAL ＆ BIOLOGICAL LABORATORIES CO., LTD) to purify each HA-tagged protein. The purified proteins were evaluated by Western blotting as described below using an anti-HA antibody (HA-Tag (C29F4) Rabbit mAb #3724, Cell Signaling Technology, Incorporated. Massachusetts. U.S.). Since the protein purified by HA-IP contains HA peptide, it was further purified by Zeba Spin Desalting Columns (7K MWCO, 2 mL, Thermo Fisher Scientific Incorporated), and the purification was confirmed by Western blotting.

***Protein expression analysis by Western blotting***

Cytoplasmic proteins and HA-purified proteins was dissolved in RIPA buffer (#182-02451, FUJIFILM Wako Pure Chemical Corporation), boiled for 10 min, electrophoresed through a 10% SDS polyacrylamide gel, and electroblotted onto a PVDF transfer membrane (IPVH00010, Millipore, Billerica, MA, USA). The membrane was blocked with PBS containing 5% skim milk and 0.05% Tween 20 and incubated for 1 hour with a DDX6 antibody (GTX102795, GeneTex Incorporated, Irvine, CA, USA), an EIF4ENIF1 (4E-T) Rabbit pAb (A15175, ABclonal, Incorporated), Anti-Glyceraldehyde-3-Phosphate Dehydrogenase Antibody, clone 6C5 (Merck KGaA, Darmstadt, Germany), anti-HA antibody (HA-Tag (C29F4) Rabbit mAb 3724, Cell Signaling Technology, Incorporated) diluted 1:500 with blocking buffer. After washing, the membrane was incubated with a 1:5000 dilution of horseradish peroxidase-linked goat anti-rabbit IgG (7074, Cell Signaling Technology, Incorporated, Danvers, Massachusetts, USA) in blocking buffer. Subsequently, the blots were developed using an enhanced chemiluminescence detection kit substrate (#1705060, Bio-Rad Laboratories Incorporated), and the protein bands were visualized using a VersaDoc system (Bio-Rad Laboratories Incorporated). The original immunoblot data were presented in Fig. S2 and Fig. S3.

***Subcellular localization of PB using cellular immunocytochemistry***

Cells were fixed with 4% PFA at room temperature for 10 min and permeabilized with 0.1% Triton X-100 at room temperature for 20 min in the presence of a protein-blocking solution consisting of PBS supplemented with 5% normal goat serum (X090710-8, Agilent Technologies Incorporated., Santa Clara, CA, USA). The cells were incubated overnight with a DDX6 antibody (1:200, GTX102795, GeneTex Incorporated) in PBS at 4°C. The cells were washed extensively in PBS and incubated at room temperature for 30 min with a secondary antibody. The nuclei were counterstained with 4′,6-diamidino-2-phenylindole (DAPI; diluted 1:500, 5748, FUJIFILM Wako Pure Chemical) in PBS at room temperature for 30 min. To prevent fading during microscopy, the cells were mounted in DakoCytomation fluorescent mounting medium (#S302380-2, Agilent Technologies Incorporated). Immunofluorescence images were visualized and recorded using a Biorevo BZ-9000 fluorescence microscope (Keyence Corporation, Osaka, Japan).

***Changes in the number and dynamics of EGFP-DDX6 in cells***

A549 EGFP-DDX6 cells were seeded at 5 × 10^3^ per well in 96 well plates. The next day, the small molecule compound (Oita University Institute of Advanced Medicine, Incorporated) or vehicle control (DMSO, FUJIFILM Wako Pure Chemical Corporation) was added to the cells and the cells were incubated. After 48 hours of exposure, the cells were incubated with Cellstain Hoechst 33342 solution (DOJINDO LABORATORIES. Kumamoto, Japan) at 37ºC for 30 min. These cells were immediately photographed for nuclei and EGFP foci in the cells with In Cell Analyzer 2200 and counted with In Cell Analyzer Workstation 3.7 (GE Healthcare Life Sciences, Pittsburgh, PA). Data were evaluated as the number of PBs per cell by dividing the total number of EGFP foci by the number of nuclei.

***Quantification of adipogenic differentiation induction using Oil Red O staining***

Fifteen micrograms of oil red O (ORO) powder (1320-06-5, Sigma-Aldrich) was dissolved in 30 ml of 100% isopropyl alcohol by gentle heating in a water bath at 37°C. After dissolution, the solution was diluted with 20 ml of distilled water and filtered to remove undissolved powder. Cells in cell culture plates were washed with phosphate-buffered saline (PBS, 166-23555, FUJIFILM Wako Pure Chemical Corporation) twice, fixed with 4% paraformaldehyde (PFA, 163-20145, FUJIFILM Wako Pure Chemical Corporation) for 10 min at room temperature, and rinsed with 60% isopropyl alcohol. The samples were stained with ORO solution for 30 min at room temperature. After fat droplets in adipocytes were stained, the samples were rinsed with 60% isopropyl alcohol again. ORO-stained cells were observed, and images were captured with an IX71 inverted microscope (Olympus, Tokyo, Japan) or a Biorevo BZ-9000 fluorescence microscope. For quantification of adipogenesis, ORO was dissolved in 100% isopropyl alcohol. The absorbance of each sample was measured by an iMark microplate reader (Bio-Rad Laboratories Incorporated.) at 492 nm.

***Gene expression analysis***

Total RNA from each sample was extracted using Trizol (Thermo Fisher Scientific Incorporated) and a Directzol RNA MiniPrep Kit (Zymo Research, Irvine, California, USA) with DNase I, according to the manufacturer's instructions. To perform the quantitative reverse transcriptase polymerase chain reaction (qPCR), 100 ng of total RNA was reverse-transcribed using the PrimeScript RT reagent Kit (Takara Bio Incorporated., Shiga, Japan) and KAPA SYBR FAST qPCR Kit Master Mix (2×) Universal (KAPA BIOSYSTEMS, Boston, MA, USA) according to the manufacturers’ recommendations. qPCR was performed using the CFX Connect Real-Time PCR Detection System (Bio-Rad Laboratories, Incorporated., CA, USA). All reactions were performed in triplicate. Calculations were automatically performed by fluorescent quantitative detection system software (CFX96 system, Bio-Rad Laboratories, Incorporated.). PCR primers were listed in Supplementary Table 2.

***Intracellular staining with Phalloidin-FITC***

Intracellular actin filaments were stained with Phalloidin-FITC (ab235137, Abcam public limited company, Cambridge, UK) for fluorescence immunostaining according to the manufacturer’s instructions. Stained actin filaments were evaluated using fluorescence microscopy and FACS. For fluorescence microscopy, intracellular staining with Phalloidin-FITC was performed as follows: EMT-induced A549 cells by TGFB2 (302-B2-002/CF, R&D Systems, Incorporated) were reacted with 4% PFA for 10 min at room temperature, and then with 0.1% Triton-X100 (FUJIFILM Wako Pure Chemical Corporation) for 10 min at room temperature. After washing twice with PBS, Phalloidin FITC Reagent and DAPI (diluted 1:500) were added to PBS and the cells were allowed to react for 30 min. These cells were further washed twice with PBS. Immunofluorescence images were visualized and recorded using a BIOREVO BZ-9000 fluorescence microscope (Keyence Corporation). For FACS analysis, intracellular staining with Phalloidin-FITC was performed as follows: EMT-induced A549 cells by TGFB2 (5 ng/ml) were detached by Trypsin/EDTA treatment and then fixed in 4% PFA at room temperature for 10 min. The fixed cells were reacted with 0.1% Triton X-100 for 10 min at RT. Then, the cells were washed by centrifugation twice (200 × g for 5 min, RT) with autoMACS Running Buffer (Miltenyi Biotec B.V. & Co. KG, Bergisch Gladbach, Germany), and incubated in autoMACS Running Buffer with Phalloidin-FITC (500-fold dilution), and allowed to react with the cells for 30 min at room temperature under light-shielding conditions. These detached cells were measured by Attune Flow Cytometer (Thermo Fisher Scientific Incorporated) and analyzed by Flow Jo software (v 10.6.1, Becton Dickinson & Company, Franklin Lakes, NJ, USA).

***PB sorting and RNA-seq***

Isolation and sorting of PBs were performed according to a fluorescence-activated particle sorting (FAPS)^4^. EMT-induced A549 cells were trypsinized, detached, and suspended in lysis buffer (50 mM Tris-HCl (pH7.4), 1 mM EDTA, 150 mM NaCl, 0.2% Triton X100, RNase and Proteinase inhibitor). The suspended cell solution was allowed to react at 4ºC for 20 min while cell disruption was performed using a syringe (1 stroke/min, about 20 times). The crushed cells were centrifuged at 200 × g and the supernatant solution was transferred to a fresh tube. DNase was then added to the transferred solution and allowed to react at room temperature for 30 min. After the reaction, the supernatant solution was discarded by centrifugation at 10,000 × g for 7 min, and the precipitates were dissolved in fresh Lysis buffer. The solution was analyzed by FACS and the GFP-positive fractions were sorted using SH800S. Sorted GFP-positive PBs were directly aliquoted into tubes containing TRIzol LS reagent (Thermo Fisher Scientific Incorporated) for RNA extraction using Direct-zol RNA MiniPrep Kit as described above. The quantity and quality of RNA in the samples were confirmed by an Agilent 2100 Bioanalyzer using RNA Nano Chips (Agilent Technologies). Libraries for next-generation sequencing (NGS) were constructed using a SMARTer Stranded Total RNA-Seq Kit v2 - Pico Input Mammalian (Takara Bio Inc., Shiga, Japan) according to the manufacturer’s instructions. Sequencing was performed at the Research Institute for Microbial Diseases at Osaka University with HiSeq 3000 (Illumina, San Diego, CA, USA) using the 100 bp single-end method. RNA-seq reads were aligned to the reference human genome GRCh37/hg19 using TopHat2 v2.1.0, Gene expression analysis was performed based on the fragments per kilobase million values calculated using the RNA-seq data by Cufflinks v2.2.1. The NGS data have been submitted to the Gene Expression Omnibus online database (<http://www.ncbi.nlm.nih.gov/geo/>) under accession number GSE196532.

***Intracellular staining with Phalloidin-FITC***

Intracellular actin filaments were stained with Phalloidin-FITC (ab235137, Abcam public limited company, Cambridge, UK) for fluorescence immunostaining according to the manufacturer’s instructions. Stained actin filaments were evaluated using fluorescence microscopy and FACS. For fluorescence microscopy, intracellular staining with Phalloidin-FITC was performed as follows: EMT-induced A549 cells by Tb2 reacted with 4% PFA for 10 min at room temperature, and then with 0.1% Triton-X100 (FUJIFILM Wako Pure Chemical Corporation) for 10 min at room temperature. After washing twice with PBS, Phalloidin FITC Reagent and DAPI (diluted 1:500) were added to PBS and the cells were allowed to react for 30 min. These cells were further washed twice with PBS. Immunofluorescence images were visualized and recorded using a BIOREVO BZ-9000 fluorescence microscope (Keyence Corporation). For FACS analysis, intracellular staining with Phalloidin-FITC was performed as follows: EMT-induced A549 cells by Tb2 (5 ng/ml) were detached by Trypsin/EDTA treatment and then fixed in 4% PFA at room temperature for 10 min. The fixed cells reacted with 0.1% Triton X-100 for 10 min at RT. Then, the cells were washed by centrifugation twice (200 × g for 5 min, RT) with autoMACS Running Buffer (Miltenyi Biotec B.V. & Co. KG, Bergisch Gladbach, Germany), and incubated in autoMACS Running Buffer with Phalloidin-FITC (diluted 1:500), and allowed to react with the cells for 30 min at room temperature under light-shielding conditions. These detached cells were measured by Attune Flow Cytometer (Thermo Fisher Scientific Incorporated) and analyzed by Flow Jo software (v 10.6.1, Becton Dickinson & Company, Franklin Lakes, NJ, USA).

**Supplementary Figure legend**

Figure S1: (A) A schematic diagram of the process by which mRNA binds to a complex containing DDX6 and 4E-T and circularizes, thereby attracting RNA decay factors. (B) Design of NCluc-RecA2 DDX6 and CHD_4E-T-Cluc

Figure S2: Analysis of changes in protein expression levels in response to a small molecule inhibitor using Western Blotting.

Figure S3: Identification of expression of synthesized and purified proteins using HEK293 cells

Figure S4: Quantitative analysis of adipogenic differentiation induction using ORO staining

Figure S5: Quantitative analysis of EMT differentiation using Phalloidin-FITC. (A) Quantification of the reaction value of Phalloidin-FITC in each condition. (B) Analysis results for each EMT induction condition (n=3).

**Supplementary Table**

Supplementary Table 1: List of abbreviations and gene names

Supplementary Table 2: Primer and plasmid sequences
