# Supplementary figures and images for: A novel mRNA decay inhibitor abolishes pathophysiological cellular transition
Source: Cell Death Discov. 2022 Jun 7;8:278. doi: 10.1038/s41420-022-01076-4 (PMC9174231; doi:10.1038/s41420-022-01076-4)

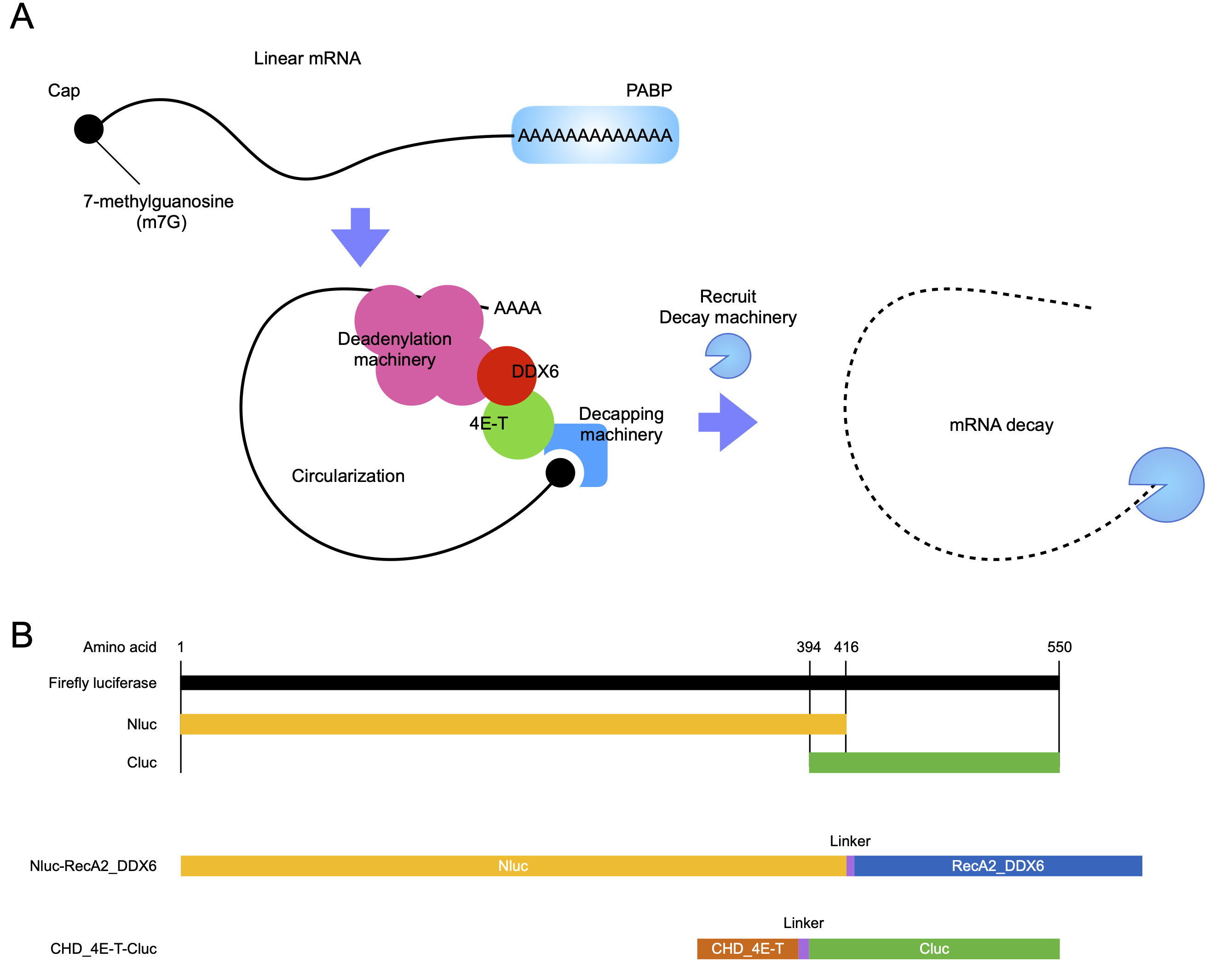

Supplement: Supplementary file 2 — Supplementary Figure 1 [file 41420_2022_1076_MOESM2_ESM.png]

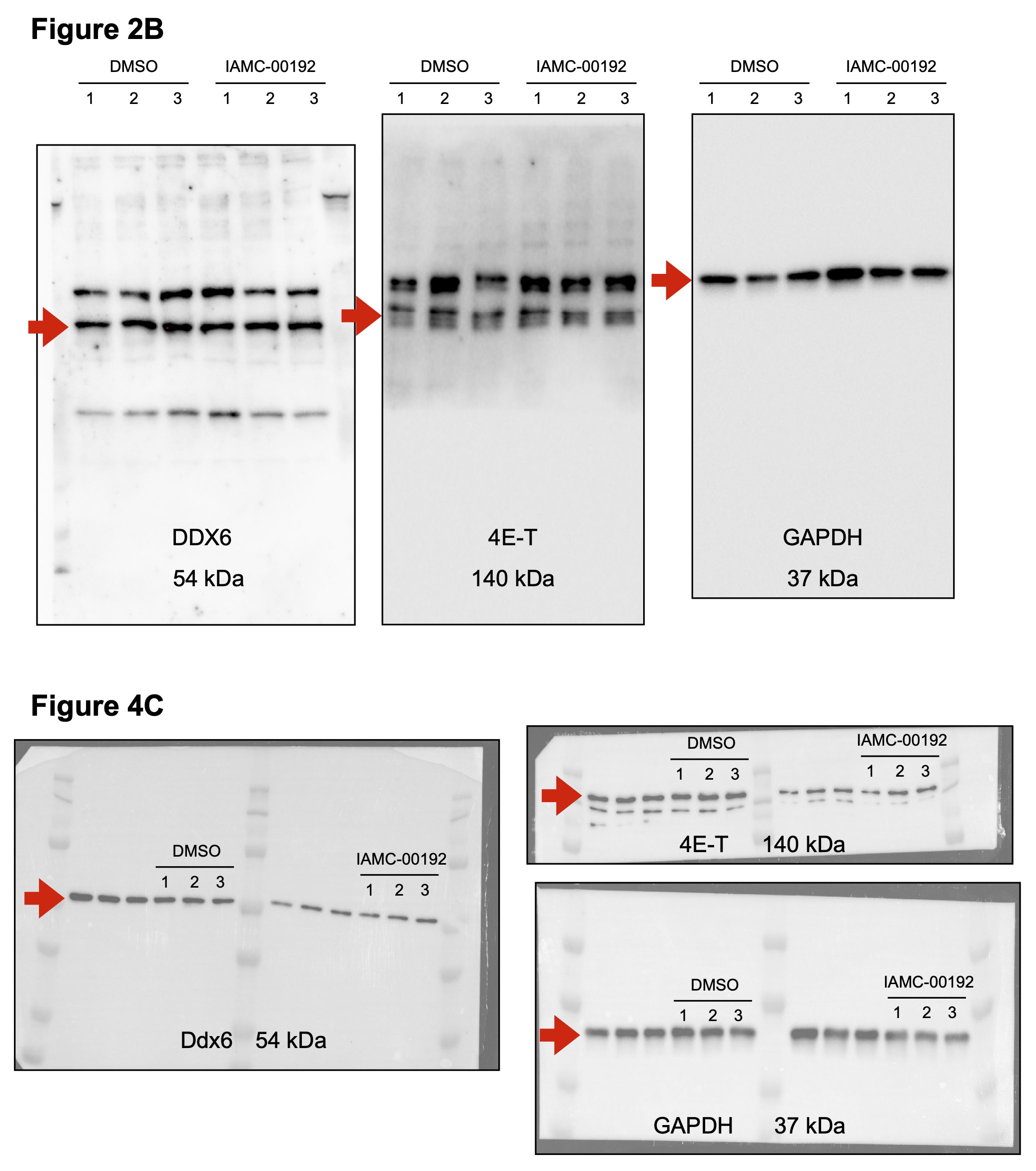

Supplement: Supplementary file 3 — Supplementary Figure 2 [file 41420_2022_1076_MOESM3_ESM.png]

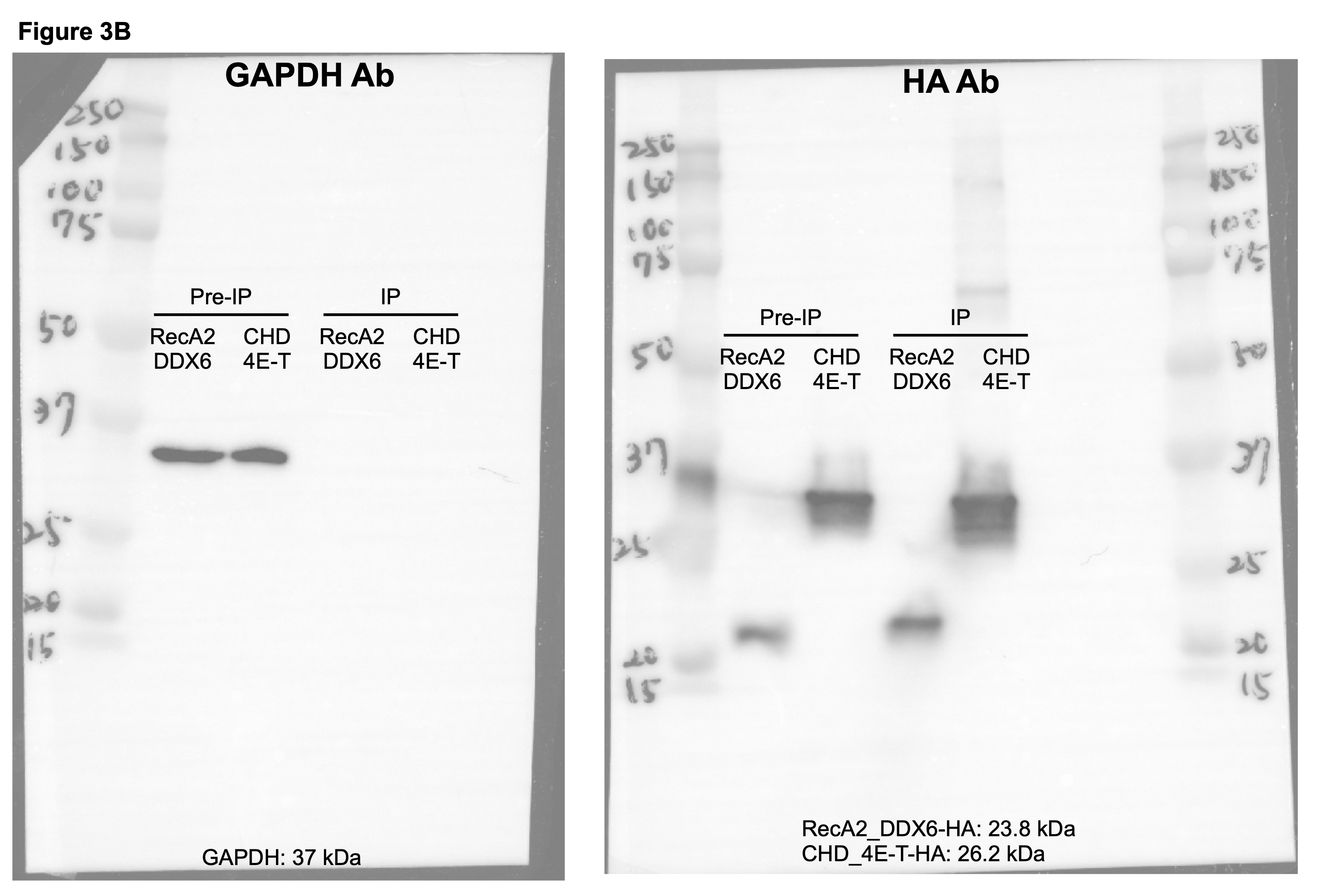

Supplement: Supplementary file 4 — Supplementary Figure 3 [file 41420_2022_1076_MOESM4_ESM.png]

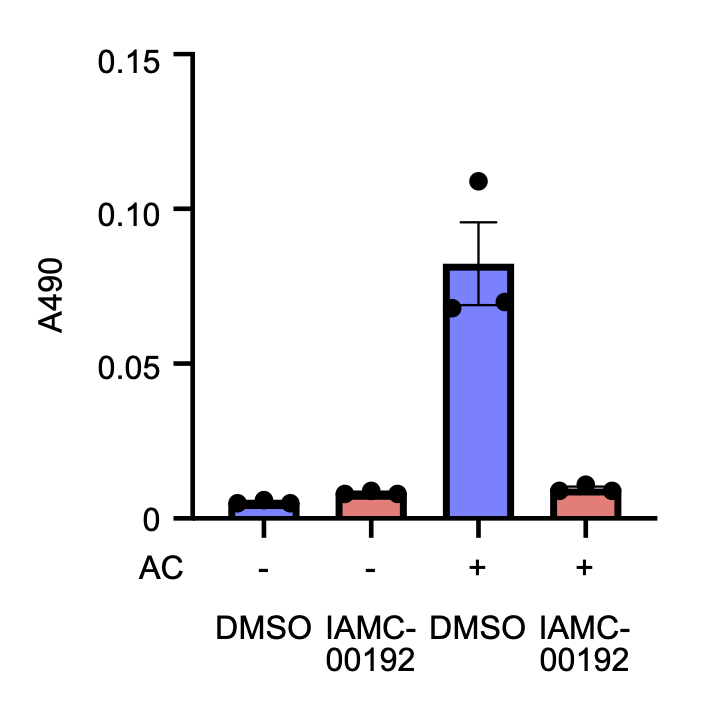

Supplement: Supplementary file 5 — Supplementary Figure 4 [file 41420_2022_1076_MOESM5_ESM.png]

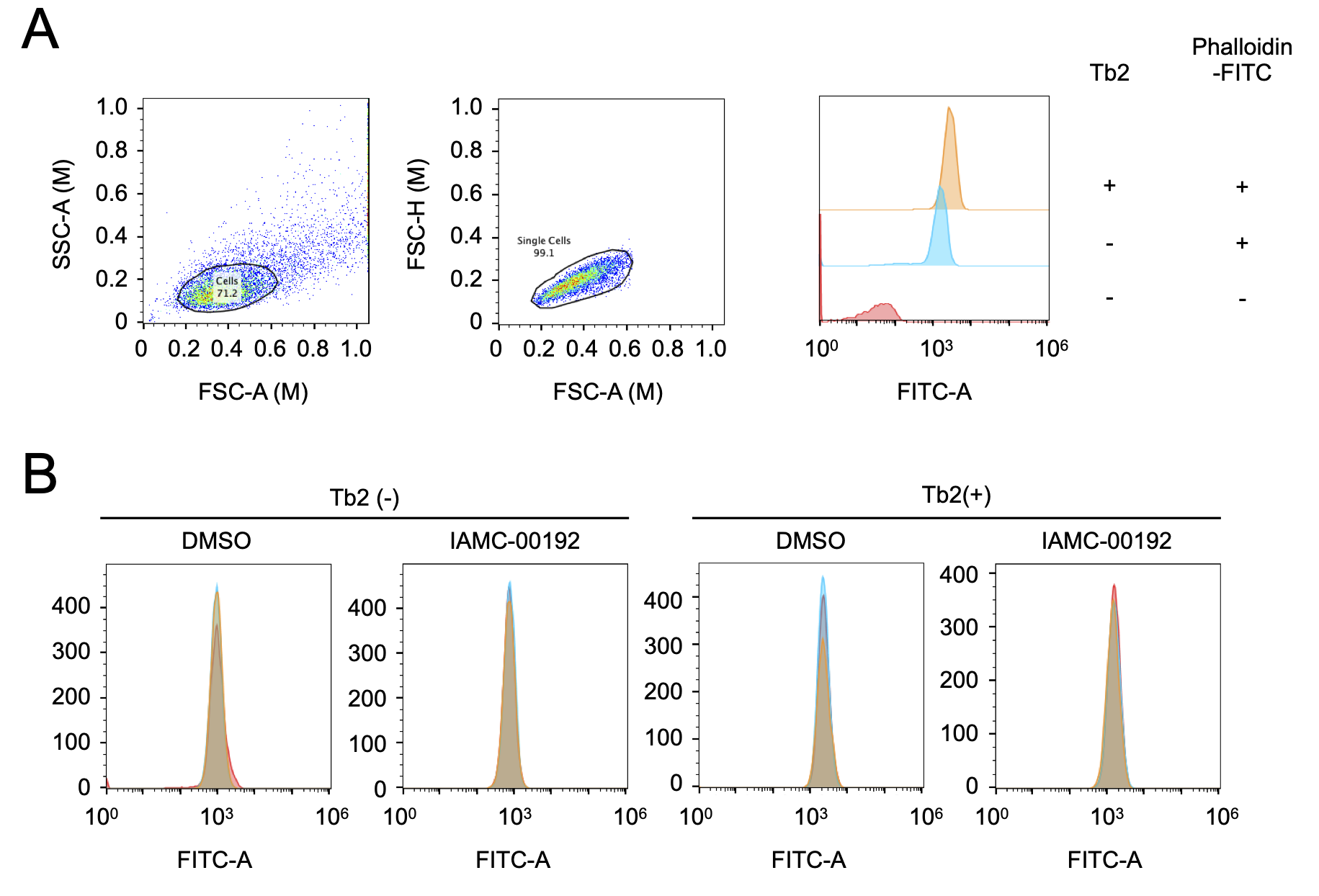

Supplement: Supplementary file 6 — Supplementary Figure 5 [file 41420_2022_1076_MOESM6_ESM.png]
